# Juvenile hormone regulates the maturation of sexually dimorphic naive ethanol olfactory preference in *Drosophila melanogaster*

**DOI:** 10.1098/rsos.242217

**Published:** 2025-08-20

**Authors:** Antonio Marini-Davis, Ryan Oliver, Bailey Godwin, Sera Chase, Ziam Khan, Fernando J. Vonhoff

**Affiliations:** ^1^ Department of Biological Sciences, University of Maryland Baltimore County, Baltimore, MD, USA

**Keywords:** sexual dimorphism, maturation, neuroendocrine, alcohol, mating

## Abstract

The molecular mechanisms underlying the maturation of innate reward behaviours remain poorly understood. We have identified a sexually dimorphic innate reward behaviour in *Drosophila melanogaster* that varies depending on the age, sex and mating status in young adults. Our results suggest that the rewarding neuropeptide *corazonin*, the transcription factor *apontic* and juvenile hormone signalling regulate naive ethanol olfactory preference responses during early stages of adulthood. Pharmacological blockade of juvenile hormone via precocene increases naive ethanol olfactory preference in females and males, which is partially phenocopied by the knockdown of juvenile hormone receptors *met* and *gce* globally in the nervous system as well as specifically in the mushroom body. The observed changes in naive ethanol olfactory preference suggest a novel role of juvenile hormone in the maturation of dimorphic ethanol behaviours during the transition from increased naive ethanol olfactory preference observed in young flies to decreased naive ethanol olfactory preference responses in older flies. Mating decreases naive ethanol olfactory preference in males, likely acting through rewarding pathways such as *neuropeptide F* and *corazonin*. Our study suggests an early evolutionary emergence of hormonal mechanisms regulating ethanol-dependent behaviours, as corazonin and juvenile hormone functionally resemble the vertebrate gonadotropin-releasing hormone and thyroid hormones, respectively. The results described here pave the way for future studies to further investigate the molecular and cellular mechanisms by which a non-reproductive, yet sexually dimorphic behaviour matures using *Drosophila* as a model. The key molecular players identified to regulate this dimorphic behaviour are conserved among species, providing fundamental knowledge to advance our understanding of sexual dimorphism and brain maturation processes relevant in numerous species.

## 1. Introduction

Brain maturation involves dynamic processes that refine connections within neuronal networks, leading to lifelong changes in structure and behaviour [1,2]. These neuronal plasticity changes are observed across species, from adolescent humans to juvenile vertebrates and invertebrates [1,3,4]. These systems are vital for the development of behaviours related to rewards necessary for survival and propagation of a species and are well conserved in even relatively simple organisms such as *Caenorhabditis elegans* [5–9]. While many rewarding stimuli have positive valence due to an organism’s experience throughout its lifetime, the valence of innate rewards such as mating is assigned by a dynamic interplay of internal and external factors, changing over time based on an organism’s physiological state and environmental conditions [10]. These complexities necessitate precise control over experimental conditions, making innate reward behaviour both challenging and essential to study. Consistently, the mechanisms underlying the refinement of innate reward behaviours during maturation at early stages of adulthood remain poorly understood. This is a particularly exciting question in developmental plasticity as early life experiences can activate fine-tuning processes and have a lasting impact on adult behaviour [11]. There is, therefore, a need to identify molecular and cellular mechanisms regulating the transition of juvenile, flexible behavioural responses to mature, predictable innate behaviours.

We use the fruit fly *Drosophila melanogaster* as a genetic model to study the maturation of sexually dimorphic innate behaviours. The mechanisms by which sexually dimorphic behaviours arise in this species have been studied extensively in neurons that develop differently between sexes as well as in neurons that regulate traditionally dimorphic behaviours such as courtship and mate searching [12,13]. For example, the transcription factors Fruitless (Fru) and Doublesex (Dsx) involved in *Drosophila* sexual determination [14,15] are expressed in some non-overlapping neuronal populations in females and males [16]. At the anatomical level, sexually dimorphic dendritic arborizations have been previously described in a subset of *dsx*^+^ neurons, the anterior dorsal neuron (aDN) cluster, that is present in the brain of both sexes [17]. aDN inputs in females are olfactory, while inputs in males are primarily visual [17]. Moreover, in males, the male-specific products of the P1 promoter of the *fru *gene (fruM) are expressed in approximately 2000 neurons that form a sex network including sensory and motor neurons [13,18–22]. fruM function is necessary for innate *Drosophila* male courtship behaviour and sufficient for some aspects of it [19,23–25]. Female-specific examples include the female-specific neuronal cluster of glutamatergic insulin-like peptide 7 (Ilp7)-expressing motoneurons that innervate the oviduct and are required for female fertility [26]. Additionally, the LSANs [27] are a pair of female-specific ascending neurons that likely transmit excitatory signals from abdominal sensory systems to the brain to modulate female receptivity and optimize reproduction [28]. Although these studies have expanded our understanding of sexual dimorphism in the context of reproduction and mating, far less attention has been given to how brain maturation is modulated by molecules and neurons that exist in both sexes to elicit non-reproductive, yet sexually dimorphic behaviours.

In natural habitats, *D. melanogaster* flies show innate sexually dimorphic attraction to ethanol-containing food [12], which is one example of an innate, non-reproductive sexually dimorphic reward behaviour observed in this species, making them a powerful model for studying particular aspects of rewarding behaviours. Besides ethanol attraction, sexual dimorphism has also been observed in additional ethanol-dependent behaviours such as ethanol-induced hyperactivity [29], sedation [29,30] and aggression (also known as post-ethanol aggression (PEA) [31]). Fruit flies evolved in an environment rich in ethanol, especially within food sources such as fermenting fruit [32]. These fermenting fruits often contain a mix of ethanol, fruit volatiles and fermentation odorants produced by *Saccharomyces* yeasts and other microorganisms [33–35]. As alcohol concentrations between 1% and 18% have been reported to be naturally produced by fermenting fruits [36,37], *D. melanogaster* is considered one of the most ethanol-resistant species among fruit-utilizing insects [35,36]. Consequently, *D. melanogaster* has become a useful model for studies of the molecular, genetic and neural mechanisms underlying ethanol-dependent behaviours [38–42]. Ecologically, it is postulated that low concentrations of ethanol are innately rewarding as they act as a cue for potential food and oviposition sites [43], which has been supported through two-choice ethanol trap assays and feeding paradigms such as the capillary feeder assay [44–46]. However, further analysis of this behaviour has revealed an array of conditions that modify a fly’s attraction to ethanol such as mating status and sex [29,47].

Although the mechanisms underlying ethanol detection in flies remain an active area of research, progress at the molecular level has been made by the identification of the odorant binding protein Obp76a, also named LUSH, whose specific binding affinity is for ethanol [48]. At the cellular level, peripheral circuits for alcohol detection in *Drosophila* involve olfactory sensory neurons (OSNs) located inside hair-like structures called sensilla that express specific olfactory receptors (ORs) [49]. Whereas ethanol is known to change pheromone detection dynamics in OSN expressing Or67d, Or47b and Or83c, three additional OSN types (Or42a, Or42b and Or59b) have been proposed to detect alcohol signals from the olfactory periphery into the brain [37]. Brain centres for learning and memory include the mushroom body (MB) and the lateral horn, which are structures that receive olfactory information and are involved in alcohol-associated memories [50,51]. Whereas the establishment of alcohol-associated preference requires dopamine neurons [52], several neurotransmitters can modulate fly behaviour in response to alcohol such as serotonin, corazonin (Crz), and neuropeptide F (NPF), as well as the primary male pheromone, cVA [36,53]. It has been shown that the genetic sex of a small (<20) subset of sex-shared neurosecretory neurons, the Crz neurons, regulates sexually dimorphic sensitivity to ethanol sedation in adult flies [30]. However, while these previous works extensively characterize *Drosophila* responses to ethanol, it is still unknown whether ethanol-dependent innate behaviours change throughout early stages of adulthood.

A common process regulating brain maturation is the interplay between environmental and innate factors. Hormones, such as androgens in mammals or ecdysone in insects, are molecular orchestrators linking global changes and acting in various tissues [54]. The sesquiterpenoid juvenile hormone (JH) has been extensively studied for its role in insect metamorphosis [54,55], but a few recent studies have explored its role in the adult nervous system, including maturation processes [56–60]. In *Drosophila*, dynamic titres of JH peak at eclosion and decrease after 2–3 days into adulthood [4,61,62]. Interestingly, the large peak of JH just after eclosion is higher in females than in males, which falls more rapidly and to a lower level in females than in males. Consequently, males maintain higher JH levels per fresh weight than females from 24 to 72 h after eclosion, suggesting a sexually dimorphic role of JH signalling [62]. In fact, in females, JH is involved in the maturation of receptivity by inducing sex pheromone production as well as in vitellogenesis and yolk protein uptake [63], whereas in males, JH is implicated in courtship behaviour [59,60,64,65] as well as in accessory gland protein synthesis [66,67]. However, whether JH regulates non-reproductive, sexually dimorphic reward behaviours in adult flies remains a largely unexplored question. JH is functionally analogous to thyroid hormones (THs) in vertebrates [61], although additional experimental evidence would help confirm the level of conservation. THs have been associated with neuropsychiatric conditions involving maladaptive reward behaviours [68,69]. While extensive research has characterized THs as key players in these behaviours, the mechanisms by which it does so are still poorly understood. By elucidating the molecular pathways influenced by JH, our results may provide fundamental information to identify conserved hormonal mechanisms underlying predisposition to harmful or addictive substances and inform therapeutic strategies.

Here, we tested >57 000 flies and have identified an early time window in which young naive flies display increased olfactory attraction to moderate ethanol sources. This naive ethanol olfactory preference (NEOP) changes over time, as older flies no longer show ethanol attraction but rather aversion. Additionally, we identified sex differences in NEOP responses between males and females that are modulated by mating status and JH-dependent signalling. We find that JH-dependent signalling is required in the nervous system, specifically in the mushroom bodies, for NEOP modulation in young flies. At the molecular level, our data also indicate that blockade of Crz, its receptor CrzR and the transcription factor apontic leads to changes in NEOP, which sexually dimorphically depends on the presence of JH. Taken together, our results suggest a novel role of JH in the maturation of dimorphic ethanol behaviours in adult flies.

## 2. Results

**Figure 1.**
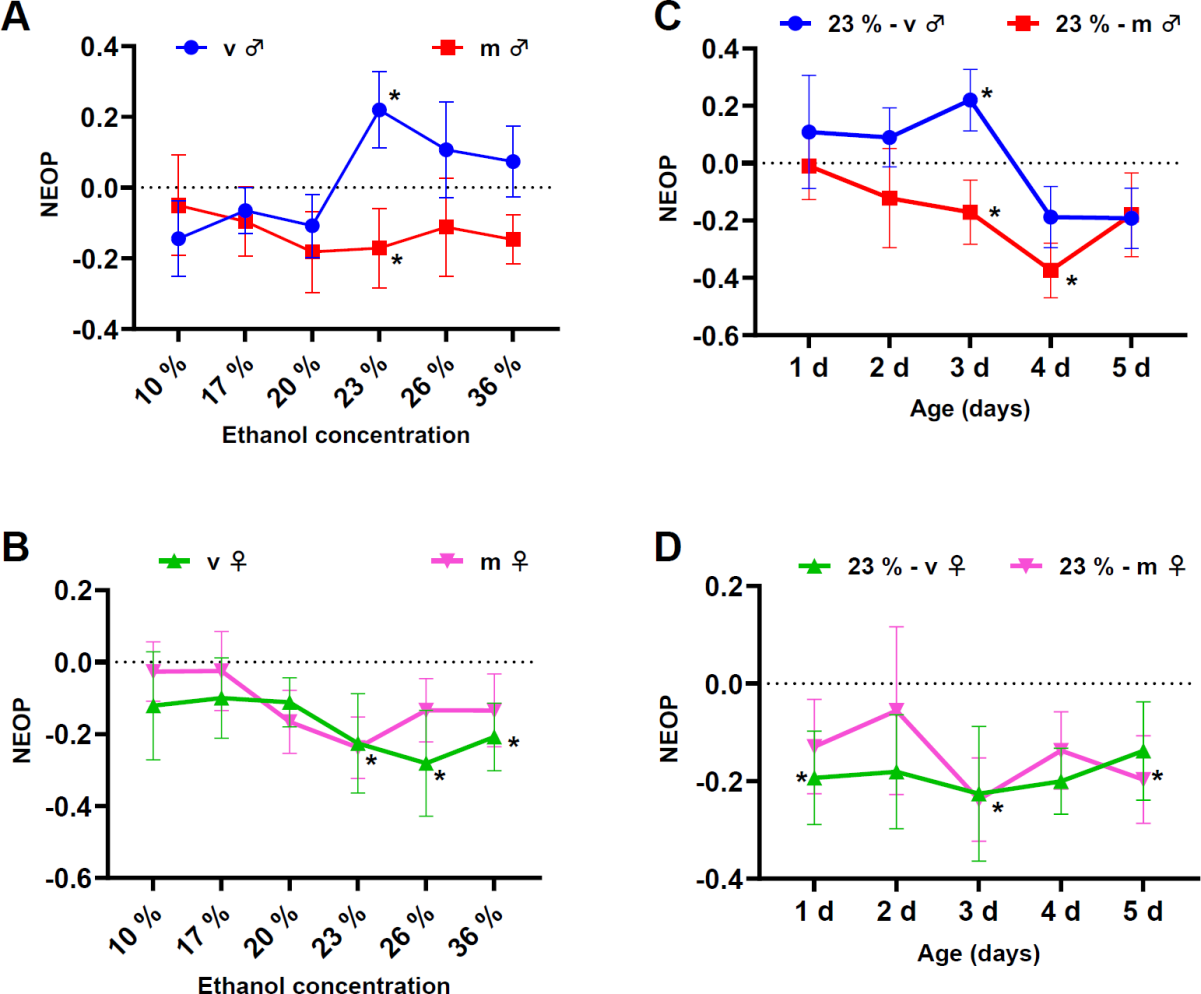
Naive ethanol olfactory preference (NEOP) responses depend on age, sex and mating status in *Drosophila*. (A,B) NEOP index for 3-day-old wild-type (DGRP-774) virgin or mated males (A) and females (B) at various (0–36%) ethanol concentrations. (C,D) NEOP for virgin or mated wild-type (DGRP-774) males (C) and females (D) given the choice between 23% ethanol and 23% water. Data are shown as mean ± s.e.m., with the following sample sizes: (A) virgin males: 30, 19, 30, 20, 29, 20,16 runs; mated males: 36, 12, 12, 15, 28, 12, 22 runs; (B) virgin females: 30, 10, 18, 12, 11, 10, 16 runs; mated females: 30, 20, 17, 12, 19, 16, 18 runs; (C) virgin males: 12, 24, 29, 11, 14 runs; mated males: 25, 16, 28, 27, 11 runs; (D) virgin females: 21, 14, 11, 13, 16 runs; mated females: 16, 16, 19, 22, 22 runs, respectively. Each experimental group was compared to a 50% neutral baseline for statistical significance (*p < 0.05; Wald z-test) to determine significant attraction or repulsion. Statistically significant effects of age and mating status within each sex were tested using GLMM as described in §4 (*p < 0.05, GLMM, *post hoc*, Tukey correction).

We performed olfactory preference assays to test the innate responses to various ethanol concentrations (electronic supplementary material, figure S1). We found 23% (v/v) ethanol had the most polarizing effect on males, as where virgin males were significantly attracted (figure 1A; Wald z-test, *p* = 0.011), and mated males were significantly repelled (Wald z-test, *p* = 0.043). By fitting the data to a generalized linear mixed model (GLMM), we were able to perform a *post hoc* pairwise comparison with a Tukey correction that shows that virgin and mated flies respond differently to ethanol concentration at 23% in males (*p* = 0.023) but not in females (figure 1A,B; *p* > 0.05; electronic supplementary material, figure S2), which suggests that mating status influences naive ethanol attraction only in males. As age is another factor that has been evidenced to influence reward processing [47], we also wanted to examine whether age could influence NEOP. Using the same T-maze paradigm, we revealed a significant decrease in ethanol preference in virgin males from 3 days after eclosion to 4 days after eclosion (*post hoc*, Tukey correction, *p* = 0.002). This was the largest contrast in preference between each age and was the only significant change in preference across both sexes and mating statuses based on age (figure 1C,D; electronic supplementary material, figure S2). This attraction is suppressed in older 4−5 day virgin males, suggesting a transition from (juvenile) increased NEOP to (mature) decreased NEOP. Thus, we used 3 day post-eclosion flies for the rest of the study to investigate possible explanations for this sexually dimorphic behaviour. Interestingly, coefficient of variation values and intragenotypic variability in males were higher for most conditions than the ones observed in females (electronic supplementary material, figures S8 and S9). These results suggest that behavioural variability in ethanol-dependent responses is differentially affected in a sex-dependent manner.

**Figure 2.**
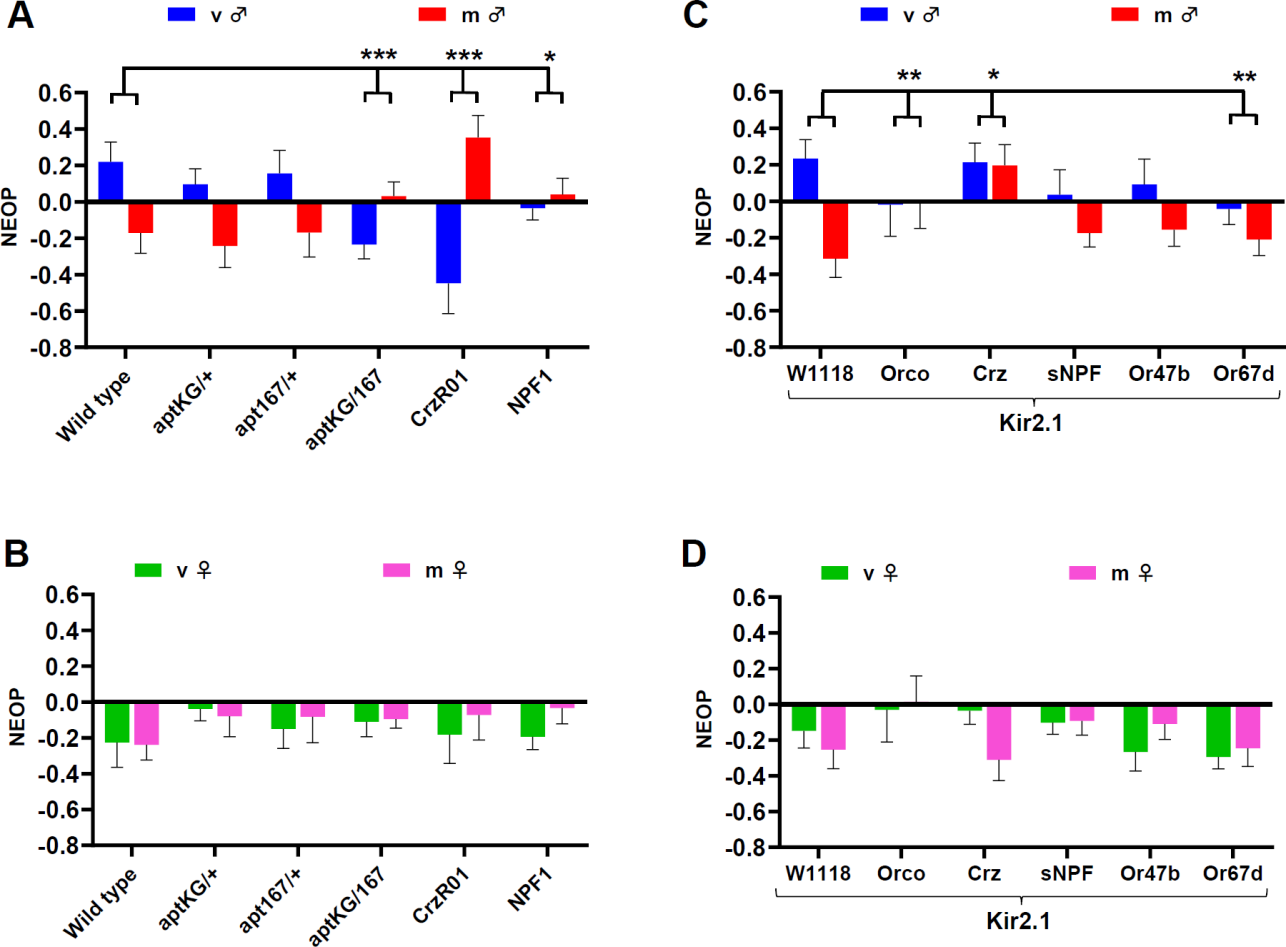
The neuropeptide *corazonin *and the transcription factor *apontic *regulate NEOP in males. (A,B) NEOP index for 3-day-old flies using 23% ethanol versus 23% water for various mutants or flies expressing the inward rectifying *Kir2.1* channel to electrically silence specific cell populations. Data are shown as mean ± s.e.m., with the following sample sizes: (A) virgin males: 29, 25, 15, 29, 14, 44 runs; mated males: 28, 12, 15, 27, 18, 38 runs; (B) virgin females: 11, 23, 16, 25, 19, 35 runs; mated females: 19, 12, 12, 28, 21, 33 runs; (C) virgin males: 15, 15, 16, 13, 23, 33 runs; mated males: 21, 24, 20, 19, 19, 24 runs; (D) virgin females: 11, 13, 16, 20, 20, 22 runs; mated females: 16, 17, 17, 24, 15, 17 runs, respectively. The following control groups were used for GLMM-based statistical comparisons: *DGRP-774* at 23% ethanol for (A,B) and W1118 × UAS-Kir2.1 at 23% ethanol for (C,D) (*p < 0.05, GLMM, *post hoc*, Holm correction).

To gain insight into the mechanism underlying NEOP, we tested molecules that we previously showed to regulate dimorphic ethanol behaviours such as the transcription factor *apontic *(*apt*) and the neuropeptide *Crz *[30], which acts via its G-protein-coupled receptor *CrzR *[70]. We tested this hypothesis by quantifying responses of *CrzR*^*01*^ mutant flies in our T-maze paradigm and observed a strikingly significant inversion in NEOP responses in males (*post hoc*, Holm correction, *p* = 0.00014 in virgins, *p* = 0.0048 in mated) that was dependent on mating status (GLMM, *p* < 0.000001), which was not present in females (GLMM, *p* = 0.44). Similarly, NEOP responses were differently affected in *apt*^*167/KG05830*^ heteroallelic mutant males based on mating status (figure 2A,B; electronic supplementary material, figure S3A,B; GLMM, *p* = 0.0003), with virgin males experiencing a significant reversal of NEOP response (*post hoc*, Holm correction, *p* = 0.0018). These effects were not observed in females (GLMM, *p* = 0.70). As NPF neurons are part of the fly reward network and involved in goal-directed behavioural drives [12,27,47], we tested *NPF*^*1*^ mutant flies and observed that mated and virgin males respond differently from wild-type flies based on mating status (GLMM, *p* = 0.017), with virgin males experiencing a significant change in NEOP (*post hoc*, Holm correction, *p* = 0.039).

In order to identify neuronal networks regulating NEOP, we expressed the inwardly rectifying potassium channel *Kir2.1* [71] to silence various candidate cell populations (figure 2C,D). We confirmed that NEOP responses obtained in our assay mostly rely on olfactory cues, as random choices are observed in anosmic flies (that express UAS-Kir2.1 via orco-GAL4) showing a shift of NEOP based on mating status (figure 2C,D; electronic supplementary material, figure S3C,D; GLMM, *p* = 0.008), which was a significant change in NEOP in mated males (*post hoc*, Holm correction, *p* = 0.042). These changes were not observed in females (GLMM, *p* = 0.15). Our result is in agreement with previous studies showing that anosmic adult flies were no longer attracted to ethanol [37,72].

Consistent with our *CrzR*^*01*^ results, silencing of Crz-expressing neurons caused mated males to have a strong significant increase in NEOP response, which phenocopies virgin male response (figure 2C; electronic supplementary material, figure S3C; *post hoc*, Holm correction, *p* = 0.0016). However, in contrast to *CrzR*^*01*^ mutants, Crz neuron silencing in virgin males did not affect NEOP (figure 2C). This difference may suggest the regulation of NEOP by different pathways either via synaptic release from Crz neurons or via interactions of the GPCR CrzR in downstream neurons. As some Crz neurons express the *short NPF* (*sNPF*) [12], we tested the role of sNPF neurons in NEOP regulation, but their silencing led to similar trends as observed in controls, maintaining positive and negative NEOP for virgin and mated males, respectively (figure 2C,D; electronic supplementary material, figure S3C,D; GLMM, *p* = 0.12). Since mating status modulates NEOP in males, we tested the role of ORs involved in female detection, including neurons expressing the ORs *Or47b* and *Or67d*, which regulate courtship [73,74] and express the sexual determination factor *Fruitless* (*Fru*) [20,24,73]. While we observed no significant changes based on mating status in females (figure 2D; electronic supplementary material, figure S3D; GLMM, *p* > 0.05), we observed an effect on male NEOP response based on mating status following Or67d silencing similar to the *orco* manipulation in males (figure 2C; electronic supplementary material, figure S3C; GLMM, *p* = 0.009).

**Figure 3.**
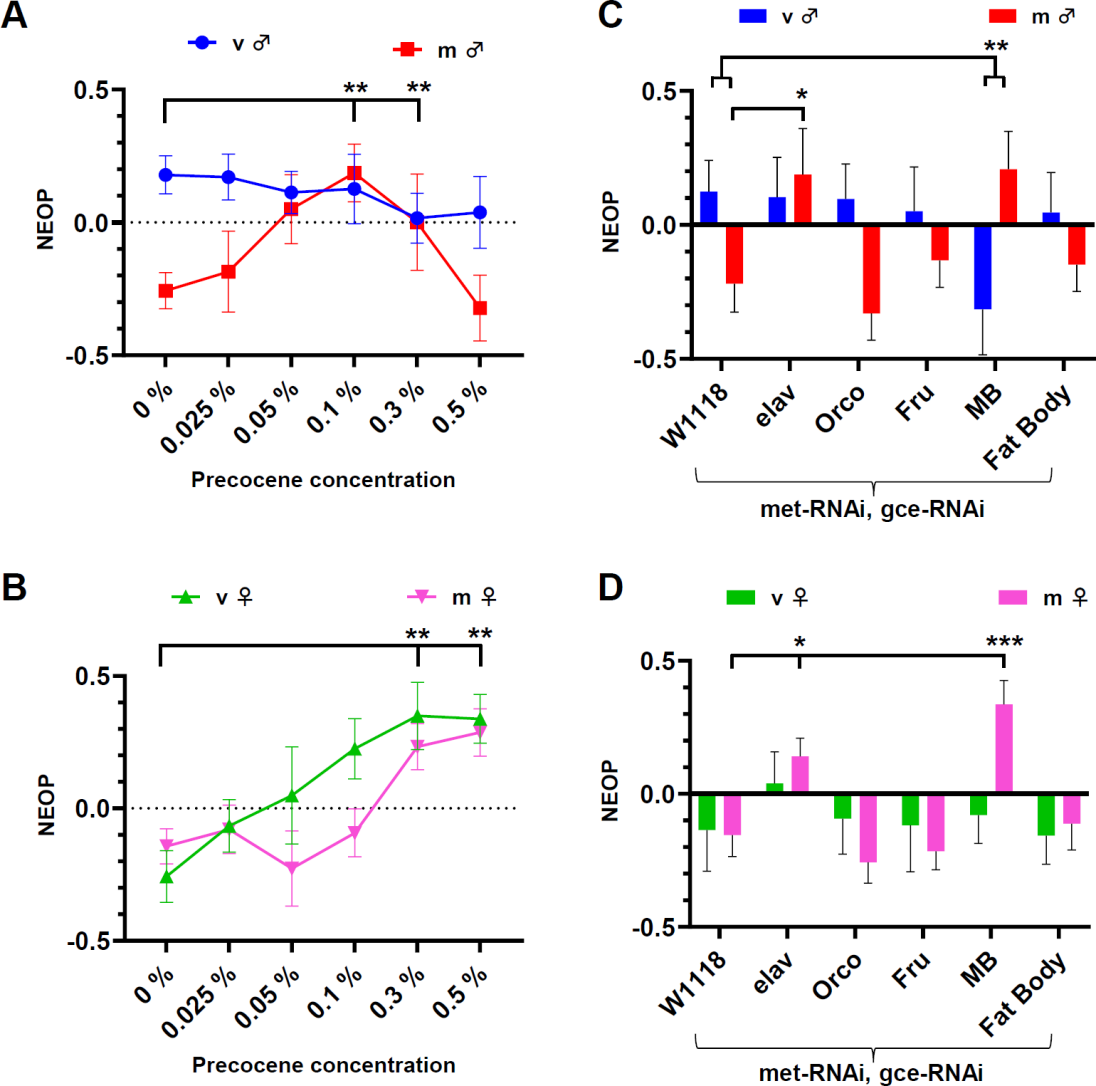
Juvenile hormone signalling regulates NEOP responses. (A,B) NEOP index for 3-day-old wild-type (*DGRP-774*) virgin or mated males (A) and females (B) fed with various precocene concentrations after eclosion. (C,D) NEOP index for various flies expressing RNAi constructs to knock down the JH receptors Met and Gce in specific cell populations (elav, pan-neuronal; orco, all olfactory neurons; Fru, Fruitless expressing neurons; MB, mushroom bodies; fat body). Data are shown as mean ± s.e.m., with the following sample sizes: (A) virgin males: 24, 10, 28, 16, 19, 14 runs; mated males: 33, 12, 13, 18, 23, 10 runs; (B) virgin females: 15, 12, 10, 11, 11, 13 runs; mated females: 18, 11, 11, 21, 15, 20 runs; (C) virgin males: 20, 18, 18, 12, 12, 15 runs; mated males: 42, 15, 24, 26, 24, 33 runs; (D) virgin females: 18, 24, 20, 14, 16, 19 runs; mated females: 41, 40, 34, 28, 35, 32 runs, respectively. The following control groups were used for GLMM-based statistical comparisons: DGRP-774+0% precocene at 23% ethanol for (A,B), W1118 × UAS-met, UAS-gce at 23% ethanol for (C,D) (*p < 0.05, GLMM, *post hoc*, Holm correction).

As both ORs have been studied for the regulation of pheromone sensitivity in a JH-dependent manner [75], we tested the role of JH in NEOP regulation. JH levels decrease after 2–3 days into adulthood [4,61], which also overlaps with the time window of NEOP maturation we have identified. We first blocked JH pharmacologically by exposing adult flies to various concentrations of precocene (a natural product that induces necrosis of the JH-producing gland *corpus allatum* [76,77]) immediately after eclosion (figure 3A,B). In males, we observed a significant interaction between mating status and precocene at 0.1% (figure 3A; electronic supplementary material, figure S5A; GLMM, *p* = 0.002) and 0.3% (v/v) (GLMM, *p* = 0.003). At these concentrations, mated males seem to phenocopy virgin male behaviour, becoming more attracted to ethanol. In females, we observed a significant effect of precocene independent of mating status at 0.3% (figure 3B; electronic supplementary material, figure S5B; GLMM, *p* = 0.007) and 0.5% (GLMM, *p* = 0.003). Thus, the results of JH blockade in young females and mated males suggest the appearance of the juvenile increased NEOP, indicating a novel role of JH in dimorphic ethanol behaviours in adult flies.

We next sought to identify the cell populations acting downstream of JH for NEOP regulation by targeting the expression of RNAi-based genetic constructs to knock down the JH receptors *met* and *gce* (figure 3C,D). Pan-neuronal *met*/*gce* knockdown in males caused a significant shift in NEOP independent of mating status (figure 3C; electronic supplementary material, figure S5C; GLMM, *p* = 0.035), which partially phenocopies the precocene-induced NEOP shift, indicating a role of the nervous system in NEOP regulation. In females, we also observed a similar effect to precocene treatment, with significant increases in NEOP independent of mating status (figure 3D; electronic supplementary material, figure S5D; GLMM, *p* = 0.048). We concluded that this partial recapitulation in females and mated males was sufficient to conclude that one possible mechanism of JH influence on NEOP was through neurons.

**Figure 4.**
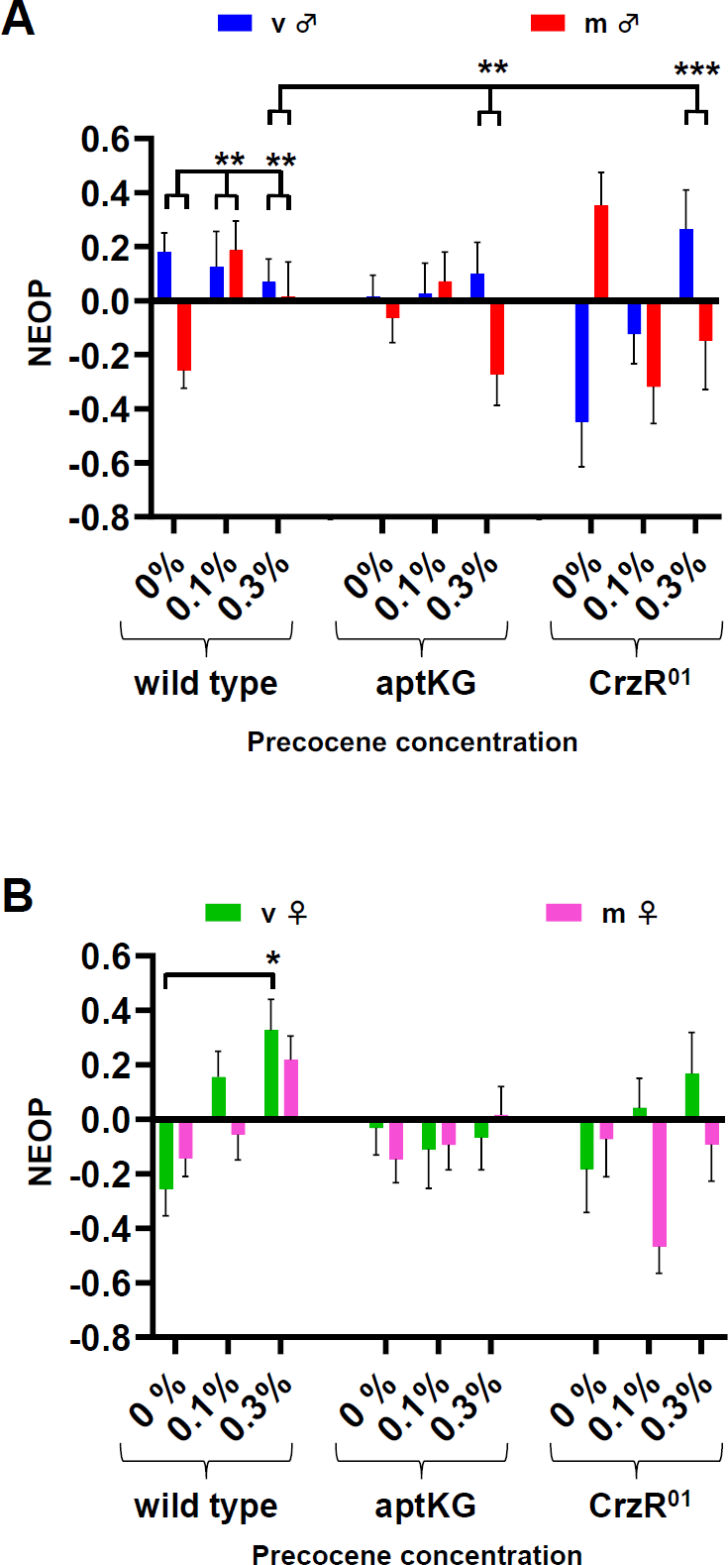
(A,B) NEOP index for virgin (V) or mated (M) wild-type (*DGRP-774*), *apontic*^*KG*^ and *CrzR*^*01*^ mutant flies fed with various precocene concentrations as labelled on the *X*-axis. NEOP index for all data based on 23% ethanol versus 23% water. Data are shown as mean ± s.e.m., with the following sample sizes: (A) virgin males: 24, 16, 19, 23, 20, 27, 14, 24, 21 runs; mated males: 33, 18, 23, 33, 30, 20, 18, 19, 12 runs; (B) virgin females: 15, 18, 21, 16, 18, 29, 19, 26, 12 runs; mated females: 18, 22, 12, 33, 30, 21, 21, 20, 10 runs, respectively. Statistical significance was calculated as described above (*p < 0.05, GLMM).

To further elucidate this mechanism, we began to investigate several potential subtypes of neurons previously evidenced to interact with JH or its behavioural correlates. *met*/*gce* knockdown in all olfactory neurons caused a minimal effect, suggesting that this behaviour is likely to be controlled by central networks rather than sensory cells (figure 3C,D). As JH has been previously described to regulate *fru* expressing neurons as well as MB neurons [61,78,79], we investigated both cell populations as potential candidate neuronal networks for NEOP regulation. Previous studies showed that Met and Gce can act as transcriptional repressors of the male isoform *fruM* in late-pupal and early-adult stages, whereas they switch to an activator after 3 days, providing further support of a role of JH in maturation processes [75]. In the MB, JH regulates the transition from an immature activity pattern observed in young (1−2 days) flies to a mature pattern observed in older 5 day flies [78]. This JH-dependent neural activity state transition in the MB is crucial for the maturation of associative learning and occurs during a similar critical period of our observed NEOP. We observed a shift of NEOP response in males expressing *met*/*gce* knockdown in the MB depending on mating status (figure 3C; electronic supplementary material, figure S5C; GLMM, *p* = 0.008) with mated males significantly increasing NEOP (*post hoc*, Holm correction, *p* = 0.032), whereas in females, we observed that mated females experienced a significant increase in NEOP (figure 4D; electronic supplementary material, figure S5D; *post hoc*, Holm correction, *p* = 0.001). From these data, we interpreted that one potential neuronal population acted on by JH to influence innate reward behaviour was within the MB.

We next tested the role of *CrzR* or *apt* signalling in increasing NEOP as observed following precocene-induced JH blockade (figure 4A,B). The result of this combination of manipulations exhibited striking sexually dimorphic effects. In males, reduction in JH titre (with precocene at 0.3% (v/v)) significantly changes NEOP responses in both *apt* (figure 4A; electronic supplementary material, figure S6A; GLMM, *p* = 0.004) and *CrzR* mutants (GLMM, *p* < 0.000001) depending on mating status. Mutants under the effects of precocene seemingly phenocopied the wild-type males of their respective mating status. A similar phenomenon was observed in females, with no significant changes from wild-type behaviour in mutants treated with precocene (figure 4B; electronic supplementary material, figure S6B; GLMM, *p* > 0.05). Although the ultimate mechanism by which JH interacts with *Crz* and *apt* is unclear, our observations suggest that these molecules interact with each other in the same molecular pathway to facilitate changes in sexually dimorphic NEOP.

## 3. Discussion

### 3.1. Factors modulating ethanol-dependent behaviours

In summary, we have identified a sexually dimorphic behaviour that matures at early stages of adulthood and is regulated by the function of various molecules including JH, Crz and Apt. Our results indicate that NEOP responses are modulated by sex, age and mating status, consistent with previous studies showing that responses to ethanol-dependent behaviours in flies are sexually dimorphic [29,30] as well as modulated by mating status, showing virgin males have stronger ethanol preference than mated flies [37,47]. The ecological relevance of the modulation of ethanol preference in flies has not been fully determined and remains a highly active research field that includes various alternative perspectives to explain the attraction of *Drosophila* towards alcohol [43]. Whereas enhanced ethanol attraction has been proposed as a self-medicating coping strategy after unsuccessful mating and mate rejection [47,80–82], a recent study has provided experimental evidence supporting the alternative hypothesis that flies are driven towards alcohols to increase their chances for subsequent mating success, a process involving a rapid amplification of fatty acid-derived pheromones [37]. Consistent with the notion that alcohol can promote mating, males exposed to alcohol to induce the PEA treatment spend less time courting and attempt to copulate earlier than alcohol-naive flies [31]. Although our results do not specifically support either of the two neuroecological interpretations for this alcohol preference, our study emphasizes the modulatory role of mating in the regulation of behavioural responses even at early stages of adulthood. In the context of maturation, our results indicate that maturation processes promote the transition of neuronal networks to a physiological state that closely represents mating-induced conditions, suggesting the possibility that this behavioural change likely provides a selection benefit for this species rather than being an irrelevant by-product in fly physiology. Future experiments can be performed to address these questions as well as to identify the mechanisms underlying the distinction between virgin and mated states, which may include experience-dependent processes modulating network connectivity or functional plasticity.

Additionally, our observed values of the ethanol preference index (signifying attraction when positive) are consistent with previous studies using food odours supplemented with ethanol [47,83]. However, our results show the strongest olfactory attraction at 23% ethanol, which is consistent with alcohol concentrations found in fermenting fruits (ranging between 1% and 18%, depending on the stage of decay and the ambient temperature levels) [36,37] as well as with a previous study showing preference for foods with 25% ethanol [44], but differs from another study showing aversion to solutions containing 23% ethanol [46]. This discrepancy could be explained by technical differences in the methods used in our study and in Ogueta *et al.* [46], including differences in container size (a 1000 ml glass beaker containing two 68 ml plastic containers [46] versus our T-maze consisting of three 46 ml plastic vials), volume of solution (1 ml apple–mango juice [46] versus our 50 μl apple juice solution), and group size (80 flies [46] versus our 8−10 flies). There are several possible mechanistic explanations for how these methodological distinctions could lead to different behavioural outcomes. A consistent behavioural output observed in our study and in previous publications including Ogueta *et al.* is that flies show aversion to relatively high ethanol concentrations. Therefore, a parsimonious explanation is that the evaporation dynamics of 50 μl of a 23% ethanol/apple juice solution on a filter paper inside of a 46 ml plastic vial with a weave cellulose acetate plug were able to lead to ethanol vapour concentrations inside the T-maze that were not perceived as excessively high by the flies in our setting. By contrast, a higher ethanol solution amount (1 ml of a 23% ethanol/mango juice solution) in a 68 ml container with a Plexiglas lid that contained a pipette tip could have facilitated different evaporation dynamics and exposure of the flies to ethanol vapour concentrations perceived as excessively high and, consequently, aversive. The mechanism regulating aversion to high ethanol concentrations could be modulated in the periphery at the level of OR neurons (supported by the lack of aversion in our experiments with anosmic flies) as well as in the central nervous system by molecular pathways involving NPF, Crz and Apt as shown in this study, or additional molecules such as alcohol dehydrogenase (Adh) [46]. A precise measurement of ethanol vapour dynamics and the regulation of fly responses via these cellular and molecular mechanisms can be the focus of future investigations. Another methodological discrepancy is the fact that mating status was not controlled in Ogueta *et al.* [46]. This allows the possible scenario that a large number of mated flies were recorded in the Ogueta *et al.* study [46], which would in fact lead to strong aversion to ethanol, ultimately consistent with our results. Whereas the hypothesis that uncontrolled mating status in prior studies would lead to reversed behaviours as a definitive cause for past discrepancies should be tested in future studies, our observations that mating status modulates ethanol preference responses in males are consistent with a previous study that examined changes in ethanol consumption [47]. Similarly, whereas most ethanol studies in flies have been conducted using only males, attraction of female flies to relatively low ethanol concentrations has been reported [46], which contrasts our observations that females show aversion to ethanol odour in most of our experimental conditions. One possible explanation is that the experimental settings in previous studies involved foods containing relatively low ethanol concentrations, which contained 5% ethanol or less [32,37,43,84], whereas our experimental set-up starting at 10% ethanol did not allow flies to detect small concentrations of ethanol due to the vaporization dynamics and the relatively small volume used in this study. Future studies can be conducted to assess and improve the technical limitations of the experimental set-up for small ethanol concentrations.

Moreover, one factor previously described to influence behavioural responses in flies is intragenotypic or interindividual variability [85,86]. This variability refers to stable behavioural differences observed between individuals despite having the same genetic background and being raised under consistent environmental factors, which has also been observed in vertebrates [87]. Whereas intragenotypic variability is unlikely to explain the contrasting preference results observed here when compared to previous studies, it may likely explain the relatively high variability levels of the behavioural responses we observed even when we tested flies from homogeneous fly populations. Despite this variability, our analysis suggests that our method of using groups of 10 flies in T-mazes reduced the effects of intragenotypic variability on the collected data, in contrast to assays using individual flies to collect data. However, behavioural variability was overall larger in males than in females. Future studies will address the biological basis for this sexual dimorphism with the expectation to identify possible anatomical or functional differences between the sexes underlying highly regulated behavioural outputs.

In mice and rats, females are known to consume more ethanol than males, a behaviour that is known to be regulated by sex chromosomes and gonadal hormones [88–90]. Consistently, the relationship between ethanol-dependent behaviours and THs as well as gonadotropin-releasing hormone in rodents and humans is well documented [69,91,92]. Therefore, our study suggests that some of these molecular mechanisms emerged earlier in the evolution of ethanol-dependent behaviours, as Crz and JH are functionally analogous to the vertebrate gonadotropin-releasing hormone (GnRH) [12,93] and THs [61], respectively.

### 3.2. Sexually dimorphic molecular regulation of maturation

Our results suggest that JH signalling at early-adult stages is required to gate the transition from juvenile (increased NEOP) to mature (decreased NEOP) behaviours during the first days of adulthood. This early-adult time interval is consistent with previous studies describing it as a critical period for memory enhancement as well as male courtship learning and modulation [4,58,61,78]. While the ethological relevance of this newly identified phenomenon is currently under investigation, we hypothesize that reward networks need to be activated at early stages (e.g. during the first 3 days of adulthood) to trigger plasticity and maturation processes to provide a selection benefit as discussed above. Consistently, factors known to represent rewarding valences in the fly nervous system, such as mating and ethanol exposure [47], represent strong candidates to regulate the activation of such maturation processes.

As NEOP responses are modulated by mating status in males, we hypothesize an interaction between mating-induced processes and JH signalling for maturation. Previous studies have described a link between mating and JH. *Drosophila* females adjust their internal state and behaviour after mating [27], including a transient increase in JH levels via the sex peptide transferred during mating, causing female gut remodelling [94]. The interaction between mating and JH in *Drosophila* males is less understood, but *Drosophila* males are expected to replace accessory gland proteins after mating, whose synthesis is JH-dependent [67]. Consistently, one hypothesis involves the possibility that JH blockade reduces mating success by blocking the production of many major seminal fluid components in the male accessory gland. Future studies will investigate the interactions between mating and dynamic JH levels in both females and males and the consequences of their dysregulation to identify components of the pathway that may be regulated in a sexually dimorphic manner.

Our results show that Crz and CrzR manipulations affected males, but not females. In males, activation of Crz is sufficient to induce appetitive learning and to induce ejaculation [12,95,96]. Our data are consistent with a model where mating, Crz/CrzR activation and dynamic JH levels decrease NEOP as observed in older, mature males. Consistently, deprivation of mating, Crz/CrzR inhibition and blockade of JH levels lead to increased NEOP as observed in younger, immature males. Activation of Crz neurons is considered rewarding, decreases ethanol consumption and increases *NPF* transcripts [95]. Thus, we hypothesize NPF neurons to be acting downstream of Crz neurons for NEOP modulation, as NPF neurons are known to interact with mating [47] and JH signalling [4]. Accordingly, an interaction between JH and downstream neurons (potentially MB or NPF neurons) may explain the fact that maturation is observed in older, virgin males based on their decreased NEOP. Interestingly, levels of *Crz* transcripts are twofold higher in adult females than in males as reported in the Flyatlas2 dataset [30], which correlates with dimorphic differences in *NPF* transcripts, being twofold higher in adult females than in males, as well as the large peak of JH just after eclosion that is higher in females than in males as described above [62]. These differences may explain the sexually dimorphic differences observed in NEOP responses here as well as in previous ethanol-dependent behaviours [12,30].

### 3.3. Juvenile hormone as a driver of mature ethanol behavioural phenotypes

We observe that blockade of JH at early-adult stages prevents the transition to mature behaviours, prolonging ethanol attraction in older flies, likely by the inhibition of JH-dependent maturation processes. This recapitulates earlier studies that have identified this early-adult time interval as a critical period for memory enhancement as well as male courtship learning and modulation [4,58,61,78], supporting a sexually dimorphic role of JH signalling. For example, in young males, JH coordinates courtship activity with reproductive maturity by modulating Or47b OSN responses [65] in a process that seems to involve the upregulation of *Pickpocket 25* (*PPK25*) [97], a member of the degenerin/epithelial sodium channel family (DEG/ENaC). Whereas the molecular aspects underlying these mechanisms remain unknown, most of these maturation processes involve the JH receptors Met and Gce, which are transcription factors of the bHLH-PAS family and act in various cell populations including the fat body, mushroom bodies, olfactory and gustatory neurons, and neurons expressing NPF [4,61,75,78].

Pharmacological and genetic blockade of JH signalling at early-adult stages leads to increased NEOP in males and females, consistent with the reduction in ethanol preference by THs observed in rats [98]. The effect on NEOP is phenocopied by knockdown of the JH receptors Met and Gce in the fly nervous system specifically in the MB, suggesting an important role of this brain area in NEOP maturation. JH has been described to suppress neuronal activity in MB and NPF neurons acting via its receptors Met and Gce in 1−2 day males [4,78]. The idea that NEOP involves the JH-dependent regulation of at least two different populations may explain our observations that pan-neuronal Met/Gce knockdown closely phenocopies the effects of the precocene treatment, whereas MB-targeted Met/Gce knockdown leads to a significant shift in NEOP based on mating status in males. Future studies will test whether blockade of JH signalling in both MB and NPF neurons is sufficient to promote NEOP in virgin and mated flies as observed in our broader pharmacological (precocene) and genetic (pan-neuronal knockdown) assays. These experiments are expected to provide mechanistic insight into the JH-dependent regulation of innate behaviour maturation at the cellular level. According to the organizational-activational hypothesis [99], hormones organize neuronal networks via anatomical changes, or they activate previously established connectivity by altering neuronal activity and gene expression. Whereas the modulation of neuronal activity levels in MB and NPF neurons supports an activational role of JH in the maturation of sexually dimorphic behaviours, the identification of neuronal subpopulations regulated by JH will allow us to test for a potential JH-dependent anatomical remodelling of these neurons, supporting a possible organizational role of JH in this process.

We propose a similar JH-dependent regulation of MB or NPF neuronal activity acting via Apt crucial for NEOP maturation as recently proposed for the maturation of associative learning [78]. Other studies have shown that Apt manipulations affect sexually dimorphic ethanol sedation behaviour [30,100]. Our Reverse Transcription quantitative Polymerase Chain Reaction (RT-qPCR) data showed a higher overall *apt* expression in males than in females [30]. Apt has been established as a feedback inhibitor of JAK/STAT signalling [101], which is a well-conserved transduction pathway regulating various processes such as cell division, cell migration and immune responses. Future studies will further investigate the role of Apt and the JAK/STAT signalling pathway in NEOP regulation, in order to provide mechanistic insight into the JH-dependent regulation of innate behaviour maturation at the molecular level. These experiments aim to identify molecular pathways acting downstream of Met and Gce receptors that may ultimately regulate changes in neuronal activity or anatomical remodelling for JH-dependent regulation of innate behaviour maturation. Given the highly conserved nature of basic signalling pathways in the nervous systems of all organisms, the insight gained from our model systems approach has the potential to advance our understanding of sexual dimorphism and brain maturation processes relevant in numerous species.

## 4. Material and methods

### 4.1. Fly stocks and husbandry

The >57 600 flies tested in this study were stored at 25°C with a 12 h dark : 12 h light cycle and fed a standard molasses fly food as previously described [30]. The fly stock DGRP-774 was used as wild-type flies and for backcrosses for the *apontic* mutant lines as described before [30]. Flies obtained from the Bloomington stock centre: UAS-Kir2.1 (RRID:BDSC_6596) [71], elavC155-GAL4 (RRID:BDSC_458), elavC155-GAL4; UAS-Dicer (RRID:BDSC_25750), orco-GAL4 (RRID:BDSC_23292), Crz-GAL4 (RRID:BDSC_51976), sNPF-GAL4 (RRID:BDSC_51991), Or47b-GAL4 (RRID:BDSC_9983), Or67d-GAL4 (RRID:BDSC_9998), NPF1 (RRID:BDSC_83722), Fru-GAL4 (RRID:BDSC_30027), MB-GAL4 (201Y-GAL4, RRID:BDSC_4440), Fat body-GAL4 (Lsp2-GAL4, RRID:BDSC_6357). The *CrzR*^*01*^ mutant line was obtained from Dr Jae Park (University of Tennessee) [102]. The different alleles of the *apontic* mutant lines *apt*^*KG05830*^ and *apt*^*167*^ were obtained from Dr Michelle Starz-Gaiano (UMBC) and were described before [30,101,103]. The w; UAS-met-RNAi, UAS-gce-RNAi and w; UAS-met-RNAi, UAS-gce-RNAi; Tub-GAL80^ts^ were obtained from Dr. Sarah Leinwand (George Washington University). The latter was used to obtain males following pan-neuronal knockdown as described in [78].

### 4.2. Behavioural assays and precocene treatment

Behavioural assays were performed using a T-maze approach (modified from [104,105]) to test virgin and mated flies separated by sex in groups of 8−10 flies per run. Here, narrow polystyrene cylindrical testing tubes (25 mm diameter × 95 mm height; Genesee Scientific, cat. no. 32-116) were connected via a T-shaped barb fitting (1/4′ Natural Nylon Equal Barb Tee, Eldon James T0-4NN) with pipette tips at two ends and attached to the tubes via vial closures (bonded dense weave cellulose acetate; Genesee Scientific, cat. no. 49-102). The tip of the pipette tip was cut (internal diameter approx. 1 mm for males and 1.3 mm for females) to allow only one fly at a time to enter the vial and designed in a way that once flies have decided and entered one of the tubes, flies cannot exit the tube. Fifty microlitres of a testing (ethanol) or control (water) solution was added on each side of the T-maze on filter paper (Whatman 1, cat. no. 1001-032). Solutions of varying ethanol concentrations (0–95%) were diluted in apple juice for the testing solution, using the same amount for water and apple juice for the control solution and allowing for a direct comparison of the effects of the two solutions (e.g. 23% ethanol versus 23% water). Ethanol concentrations were chosen based on previous literature [46,47,106] to calibrate our set-up expecting relatively lower alcohol concentrations to be attractive and higher ones to be repulsive, as alcohol concentrations found in fermenting fruits range between 1 and 18% [36,37]. The ethanol–fruit juice mix was used based on the rationale that it is more likely to represent the flies’ encounters with ethanol in nature as well as on previous publications that used solutions of ethanol mixed in fruit juices [46,83,107]. Flies were not food- or water-deprived for our behavioural assays [106]. For visualization, a study published by the *Journal of Visualized Experiments* shows high-resolution videos of behavioural responses similar to the ones in our study [104]. The NEOP index was measured per maze as previously described [104,105] (NEOP= (no. flies in ethanol side − no. flies in control side)/total no. flies in maze). Each was grouped by sex, age (1, 2, 3, 4 or 5 days old) and mating status (virgin or mated). Runs where <80% flies left the middle vial were discarded or, in a few exceptions (such as manipulations of NPF and orco>met/gceRNAi that showed consistently low responses), allowed to respond with additional time and refreshed solutions. Mazes were maintained at room temperature or 25°C in the dark until fly preference was scored 24 h after maze set-up. Twenty microlitres of the noted precocene solution (diluted in ethanol) was applied to the surface of each food vial allowing solvent evaporation as previously described [65]. Flies were transferred to fresh precocene vials every 1−2 days.

### 4.3. Coefficient of variation and intragenotypic variability analysis

Coefficient of variation (CV) was calculated as the standard deviation divided by the absolute value of the mean, multiplied by 100. For the total CV analysis, all wild-type (*DGRP-774*) data from all conditions (age, mating status, ethanol concentration) were pooled by sex as one large group or separated by age and mating status for inter-group comparisons. Intragenotypic variability was estimated as described in [85]. We first extracted the dataset count (left–right choice) from wild-type flies tested at 23% ethanol, the number of runs within each dataset, and a collective probability for left versus right. Subsequently, for null hypothesis distributions, we generated one million vectors of the same length as each real trial using Python and populated it by randomly selecting runs with replacement. Both the synthetic and real data were binned and plotted.

### 4.4. Statistical analyses

All statistical analyses were performed using R (v. 4.4.1 and 4.4.2 for Windows; R Foundation for Statistical Computing) and visualized using either the ggplot2 and emmeans packages or GraphPad Prism 10.4.1 (GraphPad Software, Inc., La Jolla, CA, USA). Normality was tested using the Shapiro–Wilk test. Data are presented as bar diagrams (mean and s.e.m.) in main figures or as GLMM-fitted probability of choosing the ethanol stimulus in electronic supplementary material figures as described below. In all graphs, statistical significance was determined by at least *p* < 0.05 and is shown by an asterisk (*p < 0.05). A 95% confidence interval was used to determine statistical significance.

For the majority of significance claims made in our study, we fitted GLMMs to our data. GLMMs offered unique advantages over traditional pairwise comparisons. First, GLMMs account for hierarchically structured data, which was necessary for our experiments because approximately 10 flies were tested together in a single T-maze, violating the assumption of independent observations. By including a random effect, we accounted for the variability between T-mazes (e.g. apparatus-specific effects) outside of the primary predictors of interest.

GLMMs also allowed us to analyse individual fly responses without converting group-level data (e.g. the number of flies choosing the experimental or control stimulus) into a continuous, normally distributed dataset, such as the NEOP ratio used elsewhere in this study. Instead, we modelled individual responses using a binomial error distribution with a logit link. Additionally, similar to a multi-way ANOVA, GLMMs enabled us to investigate interactions between predictor variables, such as the effects of mating status on genetic manipulations. Sex was not included as a predictor variable to reduce model complexity, and we instead generated different GLMMs to represent each sex, which drastically lowered Akaike information criterion values and allowed more accurate model coefficients.

Following the guidelines outlined in Bolker *et al.* [108], we fitted binomial GLMMs using the lme4 package in R. We modelled the proportion of animals choosing the experimental versus control stimulus using a binomial GLMM with a response of cbind (Experimental.Stimulus, Control.Stimulus) ~ Genotype * MatingStatus + (1 | Apparatus). This structure reflects the grouped count nature of the data. A binomial error distribution with a logit link was used to fit the model. In cases where ‘GLMM’ is cited as the statistical test in the results section, these analyses were performed as described above. For the purposes of calculating significant changes caused by our predictor variables using GLMM, we arbitrarily chose the mated mating status as the reference group. Where the GLMM did not already provide the relevant contrast, we conducted *post hoc* pairwise comparisons using appropriate multiple testing corrections (Tukey or Holm) depending on the number and nature of the comparisons. For Wald z-tests, we used a null hypothesis of 50% probability of selecting the experimental stimulus.

## Data Availability

All data have been deposited using the Dryad data repository [109]. Electronic supplementary material is available online [110].
